# Development and validation of a nomogram combining pain score with laboratory indicators for predicting persistent organ failure in acute pancreatitis: a retrospective cohort study

**DOI:** 10.3389/fmed.2024.1411288

**Published:** 2024-08-06

**Authors:** Jiayu Xing, Musen Xu, Jiale Xu, Jiao Liu, Fang He

**Affiliations:** ^1^Shanxi Bethune Hospital, Shanxi Academy of Medical Sciences, Tongji Shanxi Hospital, Third Hospital of Shanxi Medical University, Taiyuan, China; ^2^Tongji Hospital, Tongji Medical College, Huazhong University of Science and Technology, Wuhan, China

**Keywords:** acute pancreatitis, persistent organ failure, nomogram, pain score, prediction model

## Abstract

**Background:**

Acute pancreatitis is an inflammatory disease that can lead to persistent organ failure (POF), which is associated with increased morbidity and mortality. Early prediction of POF in AP can significantly improve patient outcomes.

**Objective:**

To develop and validate a nomogram that combines pain score with laboratory indicators for predicting POF in patients with AP.

**Methods:**

A retrospective cohort study was conducted, including patients diagnosed with AP. Pain score and laboratory indicators were collected within the first 24 h of admission. A nomogram was developed using logistic regression models and validated in a separate cohort.

**Results:**

There were 807 patients in the training cohort and 375 patients in the internal validation cohort.Multivariate logistic regression demonstrated that pain score, serum creatinine, hematocrit, serum calcium, and serum albumin were independent risk factors for the incidence of POF in patients with AP. The area under the curve of the nomogram constructed from the above factors were 0.924, respectively. The model demonstrated good calibration and discrimination in both the development and validation cohorts.

**Conclusion:**

The nomogram had a good performance in predicting POF in patients with AP and can be used to guide clinical decision-making.

## Introduction

Acute pancreatitis (AP) is a common clinical emergency of the digestive system (1). It refers to an acute abdomen characterized by a local inflammatory reaction in the pancreas caused by abnormal activation of pancreatic enzymes that have a digestive effect on the pancreas itself and surrounding organs (2). In severe cases, it can lead to other organ dysfunction. Its typical clinical symptoms are acute onset of persistent upper abdominal pain that can radiate to the back. In AP, persistent organ failure (POF) refers to the failure of at least one major organ system for more than 48 h. In the latest Atlanta classification criteria, severe acute pancreatitis (SAP) is defined as persistent organ failure (defined as >48 h).Therefore, POF is an important determinant of the severity of AP and is also closely related to the high mortality rate of severe acute pancreatitis (3). Studies have pointed out that the mortality rate in patients with AP combined with POF is as high as 20–30% (1). The clinical manifestations of AP are unreliable and nonspecific, and its sensitivity for predicting adverse outcomes is less than 40% (4). Therefore, early assessment of whether AP patients will develop POF is crucial to improve the prognosis of AP patients and reduce mortality.

Clinically, common scoring systems used to assess the severity of AP include: Ranson’s criteria, BISAP score, modified Marshall scoring system, and SOFA score. Ranson’s criteria is one of the earliest AP scoring systems, developed by Dr. John Ranson in the early 1970s. This scoring includes laboratory parameters at admission and after 48 h. In the study by Mikó A et al., the AUC of Ranson’s criteria for predicting SAP was 0.81 (5). Gao et al. also clearly pointed out that the Ranson score has a quite high AUC (0.83) in identifying SAP (6). Venkatesh reported that the prognostic accuracy of Ranson’s criteria for predicting SAP increased from 57.3% at admission to 73.8% after 48 h (7). However, this scoring system has its limitations; it only incorporates serological indicators during data collection, with no radiological parameters included. Furthermore, calculating the score requires at least 48 h, with an accuracy rate of approximately 75% (8). However, Ong Y pointed out that the 48-h waiting time for the Ranson score is not a disadvantage, but rather an inherent advantage, as the progression of the inflammatory response can be better assessed within 48 h (9).The BISAP score is a simple and practical scoring tool used to assess the severity of illness in patients with acute pancreatitis. It aims to provide a straightforward and rapid method to predict the mortality and complication risks in patients with acute pancreatitis. The BISAP scoring system is based on the following five clinical parameters: (1) Blood Urea Nitrogen; (2) Impaired consciousness: Glasgow Coma Scale; (3) Systemic Inflammatory Response Syndrome; (4) Age; (5) Pleural effusion. The BISAP score ranges from 0 to 5, with higher scores indicating more severe illness and increased risks of mortality and complications. A BISAP score of ≥3 is a statistically significant threshold value, with the AUC for predicting SAP and mortality being 0.875 and 0.740, respectively (10). Kapadia NN et al. pointed out that the specificity of BISAP in predicting SAP is 94.62% (11). However, the assessment of the patient’s mental state in the BISAP score is often subjective. If rigorousness is pursued, it needs to be compared with the patient’s baseline mental state, but these are unknown. Additionally, pleural effusion is a complication that may develop in SAP over time and may not be present at the time of admission. The modified Marshall scoring system was originally designed to assess organ dysfunction and predict outcomes in critically ill patients in intensive care and has also been utilized for predicting SAP. The design of the modified Marshall scoring system includes the following three organ systems: (1) respiratory system; (2) circulatory system; (3) renal function. C et al. found that the modified Marshall score’s sensitivity, specificity, and AUC for predicting SAP were 83.33, 87.5%, and 0.938, respectively (12). SOFA score is a scoring tool used to evaluate the extent of organ function failure in patients, widely utilized in intensive care units. Initially proposed by Vincent and colleagues in 1996, its purpose is to describe and quantify the severity of organ failure and predict the prognosis of critically ill patients. The SOFA scoring system includes functional assessments of six organ systems: (1) respiratory; (2) coagulation; (3) liver; (4) cardiovascular; (5) central nervous; (6) renal functions. Studies indicate that the SOFA score has an AUC of 0.966 for predicting SAP (13). While the diversity of variables increases the sensitivity and specificity of the SOFA score, it complicates its use in clinical practice. These scoring systems lack sufficient research evidence for patients with POF. A recent review highlights that current early predictive markers for POF are not sufficiently accurate for individualized patient predictions. The ideal predictive marker should be applicable at the time of admission or within 24 h of onset, with an accuracy rate exceeding 90% (14).

The genesis of the pain scoring system can be traced back to the International Association for the Study of Pain (IASP) in 1979, defining pain as ‘an unpleasant sensory and emotional experience associated with actual or potential tissue damage.’ Over time, this definition has evolved to emphasize the influence of cognitive and social factors on the perception of pain. In clinical practice, pain scoring assists physicians and nurses in accurately assessing the severity of a patient’s pain, devising appropriate treatment plans, and monitoring therapeutic outcomes (15). Professional societies recommend that pain should be used as a biological indicator similar to other vital signs to jointly assess a patient’s vital status (16).

A nomogram is a statistical graphic constructed using clinical data, commonly employed to evaluate the prognosis of diseases. It generates the probability of an event’s occurrence by integrating clinical variables. Compared to traditional scoring charts, nomograms provide a more intuitive representation of the likelihood of outcomes based on various clinical indicators, facilitating numerical calculations (17).

This study aims to develop a predictive model that combines pain scoring with laboratory indicators to forecast the occurrence of POF in patients with AP. Utilizing diagnostic markers within the first 24 h of admission, the model seeks to promptly identify individuals at high risk for POF, thereby guiding clinicians to implement personalized diagnostic and therapeutic measures to improve prognosis.

## Materials and methods

### Study design and population

This study is a retrospective cohort study. It encompasses patients hospitalized and initially diagnosed with AP at Shanxi Bethune Hospital from January 1, 2012, to January 1, 2022. As a retrospective study, all data were derived from our hospital’s Hospital Information System, negating the need for informed consent from each patient (in accordance with the ethical requirements of clinical retrospective research). Moreover, the study design and implementation strictly adhered to the principles of the 1975 Declaration of Helsinki and received approval from the Ethics Committee of Shanxi Bethune Hospital (Ethical Approval Number: YXLL-2023-237). All AP patients in the study cohort were randomly divided into a training cohort and an internal validation cohort at a ratio of 7:3. Within the training set, patients were grouped based on the presence or absence of POF, and through univariate and multivariate analyses, independent risk factors for the occurrence of POF in AP patients were identified to establish a clinical prediction model, which was then validated in the validation group.

### Inclusion and exclusion criteria

Inclusion Criteria: (1) Meets the diagnostic criteria for AP; (2)Age ≥ 18 years; (3) Admission within 48 h of onset; (4) Hospitalized at Shanxi Bethune Hospital and initially diagnosed with AP.

Exclusion Criteria: (1) Incomplete clinical data or missing medical records; (2) Recurrent AP; (3) Chronic pancreatitis, trauma, or pregnancy-related pancreatitis; (4) Cancer patients; (5) Pre-existing severe dysfunction of the heart, brain, lungs, kidneys, etc., diagnosed before the onset; (6) Age < 18 years.

### Data collection and definitions

Data were retrospectively retrieved from electronic medical records, encompassing variables such as demographics: age, gender, Body Mass Index (BMI), smoking status, alcohol consumption, hypertension, length of hospital stay, and laboratory indicators within 24 h of admission: complete blood count, liver and kidney function, pancreatic function, coagulation profile, among others.

The diagnostic criteria for Acute Pancreatitis (AP) adhere to the 2012 revision of the Atlanta Classification: (1) Persistent upper abdominal pain; (2) Serum amylase and/or lipase levels more than three times the upper limit of normal; (3) Abdominal imaging findings consistent with changes of acute pancreatitis.A diagnosis of AP can be established if two of the above three criteria are met.

The diagnostic criteria for organ failure are based on the modified Marshall scoring system, where a score of ≥2 for any organ is defined as organ failure. If the condition persists for more than 48 h, it is classified as persistent organ failure.

### Statistical analysis

Statistical analyses were conducted using SPSS 26.0 and R version 4.3.2. Quantitative data following a normal distribution are presented as mean ± standard deviation, and comparisons between groups were performed using independent samples t-tests. Non-normally distributed quantitative data are expressed as median [IQR, P25; P75], with group comparisons conducted using the Mann–Whitney U test. Counts are presented as the number of cases and percentages. Comparisons between groups were made using the Chi-square (*X*^2^) test. A *p*-value <0.05 was considered statistically significant.

Variables deemed significant in the univariate analysis were incorporated into a binary logistic regression equation for multivariate analysis. The results will be used to construct a nomogram for predicting the occurrence of POF in patients with AP. The area under the curve (AUC) and 95% confidence intervals for the predictive models in the training and validation cohorts were determined using the receiver operating characteristic (ROC) curve. Additionally, calibration curves and clinical decision curves (DCA) were plotted.

## Results

**Figure 1:**
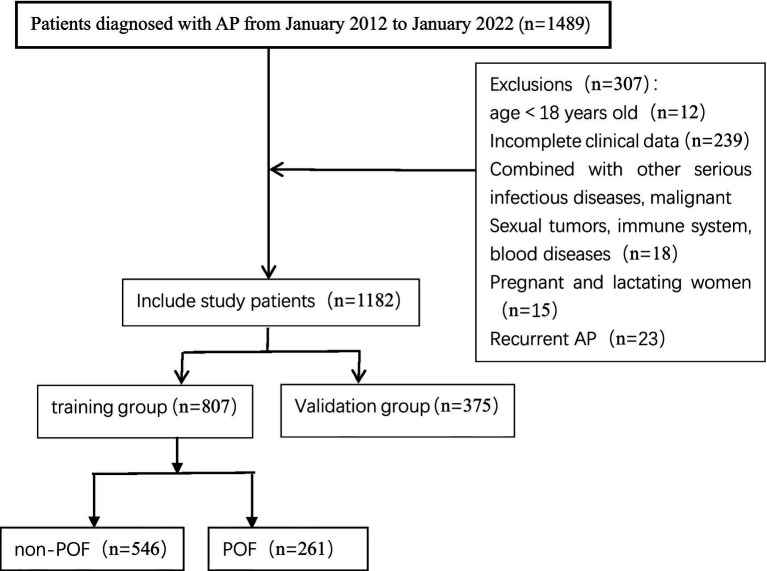
Flowchart for patient selection.

Between January 1, 2012, and January 1, 2022, a total of 1,489 patients were diagnosed with AP at Shanxi Bethune Hospital. Following the inclusion and exclusion criteria, 1,182 patients were ultimately eligible for the study (Figure 1).

### Comparison of basic characteristics and clinical parameters between patients grouped by POF

In the training set of 807 patients, they were grouped according to the occurrence of POF, with 261 cases in the POF group and 546 cases in the non-POF group (Table 1). A comparison was made between the two groups regarding basic clinical characteristics upon admission, pain scoring, and laboratory test indicators.

**Table 1:** Comparison of general characteristics and indicators of patients.

Variables	Total (*n* = 807)	non-POF (*n* = 546)	POF (*n* = 261)	*p*
Sex, *n* (%)				0.229
Female	281 (35)	182 (33)	99 (38)	
Male	526 (65)	364 (67)	162 (62)	
Age, IQR	47 (35, 61)	44 (34, 57)	52 (38, 68)	< 0.001
Time, IQR	12.53 (8.92, 17.45)	11.51 (8.28, 15.52)	15.28 (11, 23.36)	< 0.001
BMI, IQR	25.82 (23.32, 29.3)	25.65 (23.15, 28.73)	26.35 (23.71, 29.41)	0.133
Smoking, *n* (%)				0.34
No	502 (62)	333 (61)	169 (65)	
Yes	305 (38)	213 (39)	92 (35)	
Drinking, *n* (%)				0.493
No	523 (65)	349 (64)	174 (67)	
Yes	284 (35)	197 (36)	87 (33)	
Diabetes, *n* (%)				0.218
No	699 (87)	479 (88)	220 (84)	
Yes	108 (13)	67 (12)	41 (16)	
Hypertension, *n* (%)				0.112
No	599 (74)	415 (76)	184 (70)	
Yes	208 (26)	131 (24)	77 (30)	
Pain, *n* (%)				< 0.001
2	85 (11)	85 (16)	0 (0)	
3	311 (39)	298 (55)	13 (5)	
4	146 (18)	96 (18)	50 (19)	
5	131 (16)	48 (9)	83 (32)	
6	89 (11)	19 (3)	70 (27)	
7	24 (3)	0 (0)	24 (9)	
8	21 (3)	0 (0)	21 (8)	
**Liver function (IQR)**				
ALT (U/L)	45.1 (22.5, 112.25)	45.55 (23, 113.6)	44 (21, 111.66)	0.536
AST (U/L)	36.6 (22.2, 114.1)	33.45 (21.6, 114.1)	46.4 (25.2, 114.1)	0.005
ALP (U/L)	108.9 (74.8, 108.9)	108.9 (78.25, 108.9)	108.9 (70.2, 108.9)	0.05
GGT (U/L)	219.5 (78.6, 219.5)	219.5 (80.68, 219.5)	219.5 (73.2, 219.5)	0.8
ALB (g/L)	36.3 (31.3, 40.8)	36.8 (33.5, 41.7)	34 (28.7, 37.9)	< 0.001
TB (μmol/L)	22.4 (13.3, 31.75)	21.8 (13.22, 30.25)	25.1 (13.7, 35.9)	0.065
DB (μmol/L)	8.3 (3.7, 13.4)	7.4 (3.3, 13.4)	10.5 (4.9, 13.4)	0.002
TC (mmol/L)	4.57 (3.21, 4.97)	4.63 (3.37, 4.97)	4.37 (2.85, 4.99)	0.197
TG (mmol/L)	1.62 (0.89, 3.9)	1.75 (0.92, 3.9)	1.43 (0.85, 3.9)	0.414
**Kidney function (IQR)**				
Urea (mmol/L)	5.4 (4.1, 6.4)	5.15 (4, 5.8)	5.8 (4.4, 7.6)	< 0.001
SCr (μmol/L)	75.7 (62.85, 86.5)	75 (62.5, 84.4)	77.4 (63.6, 95.2)	0.005
**Pancreatic function (IQR)**				
AMY (U/L)	207.5 (80.95, 599.65)	179.8 (70.2, 537.7)	298 (105.6, 756.1)	< 0.001
LPS (U/L)	275.6 (93.6, 756.35)	237.1 (88.32, 695.65)	388.7 (111, 897.2)	0.005
Ca (mmol/L)	2.16 (2.04, 2.28)	2.19 (2.09, 2.29)	2.08 (1.93, 2.2)	< 0.001
**Coagulation function (IQR)**				
PT (s)	12.9 (12, 13.4)	12.75 (11.9, 13.2)	12.9 (12.1, 13.6)	0.008
APTT (s)	30.3 (28.4, 31.65)	30.3 (28.4, 31.7)	30.3 (28.4, 31.3)	0.321
FIB (g/L)	4.52 (3.39, 5.2)	4.4 (3.33, 5.2)	4.83 (3.58, 5.2)	0.01
**Blood routine (IQR)**				
WBC (10^9^/L)	11 (7.7, 14.3)	10.54 (7.33, 13.6)	11.9 (8.7, 15.6)	< 0.001
NEUT (10^9^/L)	9.11 (5.81, 12.09)	8.29 (5.31, 11.53)	10.14 (7.24, 13.4)	< 0.001
LYMPH (10^9^/L)	1.1 (0.78, 1.59)	1.23 (0.84, 1.71)	0.9 (0.63, 1.26)	< 0.001
RBC (10^12^/L)	4.53 (4.06, 5)	4.54 (4.08, 4.98)	4.48 (4.01, 5.06)	0.86
HCT (L/L)	0.45 (0.4, 13.95)	0.44 (0.39, 0.52)	0.48 (0.4, 34)	0.005
MCV (fL)	91.9 (89.16, 95.82)	91.8 (89.08, 95.38)	92.3 (89.3, 97)	0.055
MCH (pg)	31.3 (30.2, 32.5)	31.3 (30.1, 32.4)	31.3 (30.3, 32.7)	0.284
MCHC (g/L)	338 (332.78, 343.4)	338 (333, 344)	338.58 (332, 343)	0.52
RDW (%)	13.3 (12.5, 14)	13.2 (12.43, 13.97)	13.6 (12.8, 14.2)	0.005
PLT (10^9^/L)	202 (163, 253)	207 (170.25, 255.75)	188 (146, 242.5)	< 0.001
MPV (fL)	16.8 (16.2, 17.4)	16.7 (16.1, 17.3)	16.9 (16.5, 17.5)	< 0.001
PDW (%)	8.6 (7.95, 9.5)	8.6 (7.9, 9.5)	8.7 (8.1, 9.6)	0.038
PLR	178.26 (127.65, 258.36)	167.52 (121.09, 244.73)	204.11 (146.2, 300.79)	< 0.001
NLR	7.84 (4.36, 13.4)	6.68 (3.76, 11.35)	11.43 (6.57, 17.52)	< 0.001

IQR, median and interquartile range [P25; P75]; BMI, body mass index; WBC, white blood cell count; NEUT, neutrophil count; LYMPH, lymphocyte count; RBC, red blood cell count; MCV: mean corpuscular volume; MCH: mean corpuscular hemoglobin; MCHC, mean corpuscular hemoglobin concentration; RDW, red cell distribution width; PLT, platelet count; PDW, platelet distribution width; MPV, mean platelet volume; AST, aspartate aminotransferase; ALT, alanine aminotransferase; ALP, alkaline phosphatase; GGT, gamma-glutamyltransferase; ALB, albumin; TB, total bilirubin; DB, direct bilirubin; TC, total cholesterol; TG, triglycerides; BUN, blood urea nitrogen; SCr, serum creatinine; AMY, amylase; LPS, lipase; Ca, calcium; APTT, activated partial thromboplastin time; PT, prothrombin time; FIB, fibrinogen; PLR, platelet to lymphocyte ratio; NLR, neutrophil to lymphocyte ratio.

Basic Clinical Characteristics: There were no significant differences between the two groups in terms of gender, BMI, smoking, alcohol consumption, hypertension, etc. (*p* > 0.05). However, there were statistical differences in age, hospital stay duration, and pain scores (*p* < 0.05).

Laboratory Tests: Statistically significant differences were observed between the two groups in AST, ALB, DB, Urea, SCr, AMY, LPS, Ca, PT, FIB, WBC, NEUT, LYMPH, HCT, RDW, PLT, MPV, PDW, PLR, and NLR (*p* < 0.05). No significant differences were noted in the remaining laboratory parameters.

### Construction of predictive model

The indicators that differed between the two groups mentioned above were subjected to univariate and multivariate logistic regression analyses (Table 2). Pain scores, ALB, SCr, Ca, and HCT were identified as independent risk factors for predicting the occurrence of POF in AP.

**Table 2:** Univariable and multivariable logistic regression: risk factors for POF.

Variables	Univariable (*P* < 0.05) OR (95% CI)	*P*	Multivariable (*P* < 0.05) OR (95% CI)	*P*
Age	1.02(1.01–1.03)	<0.001		
Time	1.06(1.04–1.08)	<0.001		
Pain	4.65(3.82–5.75)	<0.001	4.58(3.67–5.84)	<0.001
AST	1(1–1)	0.03		
ALB	0.99(0.97–1.00)	0.03	0.99(0.97–1.00)	0.02
DB	1(0.99–1.01)	0.21		
Urea	1.21(1.14–1.29)	<0.001		
SCr	1.01(1.00–1.02)	<0.001	1.01(1.00–1.02)	0.02
AMY	1(1–1)	0.03		
LPS	1(1–1)	0.12		
Ca	0.03(0.01–0.08)	<0.001	0.09(0.03–0.34)	<0.001
PT	1.16(1.05–1.29)	0.004		
FIB	1.13(1.03–1.23)	0.01		
WBC	1.06(1.03–1.10)	<0.001		
NEUT	1.09(1.05–1.12)	<0.001		
LYMPH	0.48(0.37–0.63)	<0.001		
HCT	1.003(0.997–1.008)	0.16	1.007(1.00–1.01)	0.04
RDW	1.06(0.98–1.15)	0.13		
PLT	1.00(0.99–1.00)	0.002		
MPV	1.13(1.04–1.23)	0.006		
PDW	1.10(0.98–1.23)	0.11		
PLR	1.002(1.001–1.004)	<0.001		
NLR	1.06(1.04–1.08)	<0.001		

### Development of a predictive model nomogram

**Figure 2:**
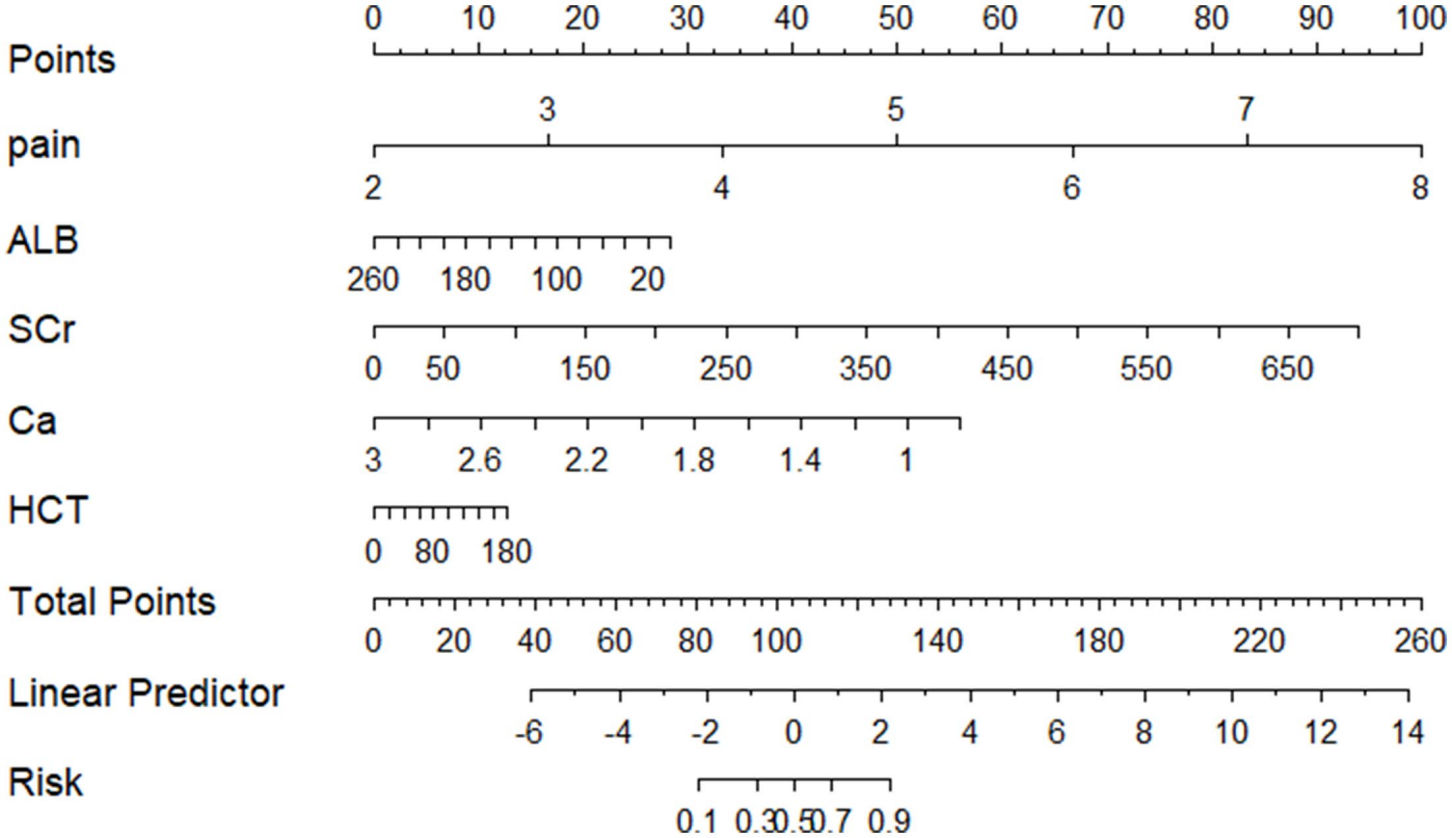
Nomogram of an early prediction model for POF in AP.

Based on these results, a predictive model nomogram was established (Figure 2). Each indicator in the figure corresponds to its test result, allowing for the determination of respective predictive scores. By aggregating the scores of each indicator, a total predictive score can be ascertained, which corresponds to the probability of POF.

### Performance evaluation of the predictive model

**Figure 3:**
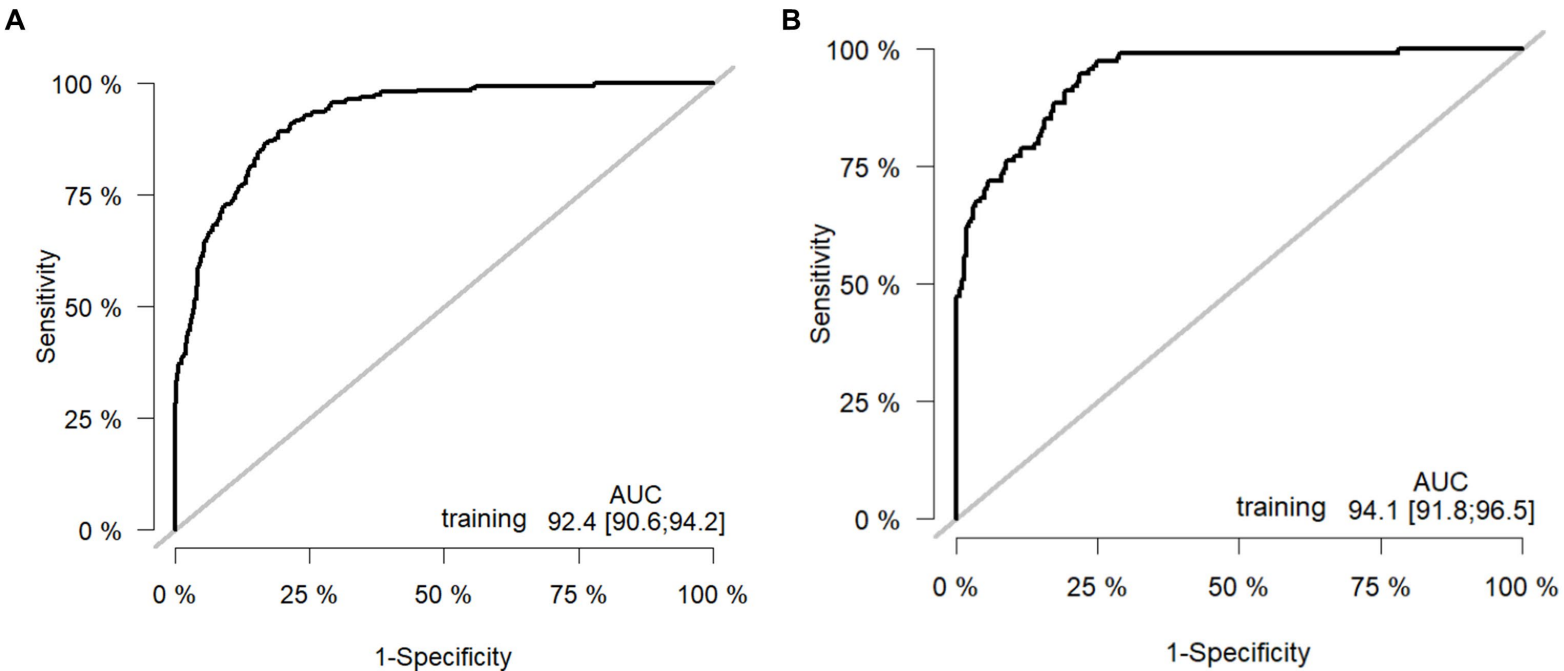
Receiver operating curves: **(A)** Training cohort. **(B)** Internal validation cohort. AUC: area under the ROC (receiver operating characteristic) curve.

**Figure 4:**
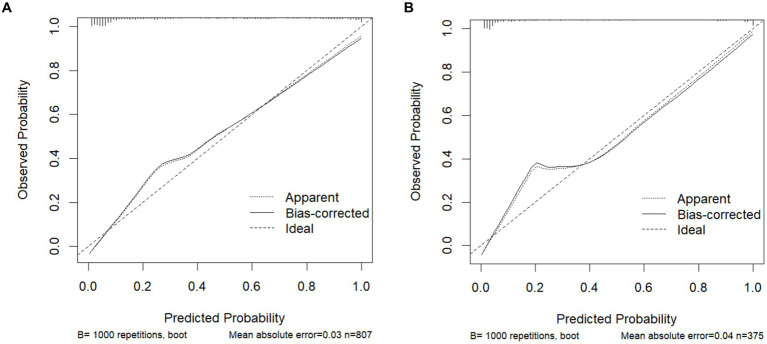
Calibration curves for predicting the POF: **(A)** Training cohort. **(B)** Internal validation cohort.

**Figure 5:**
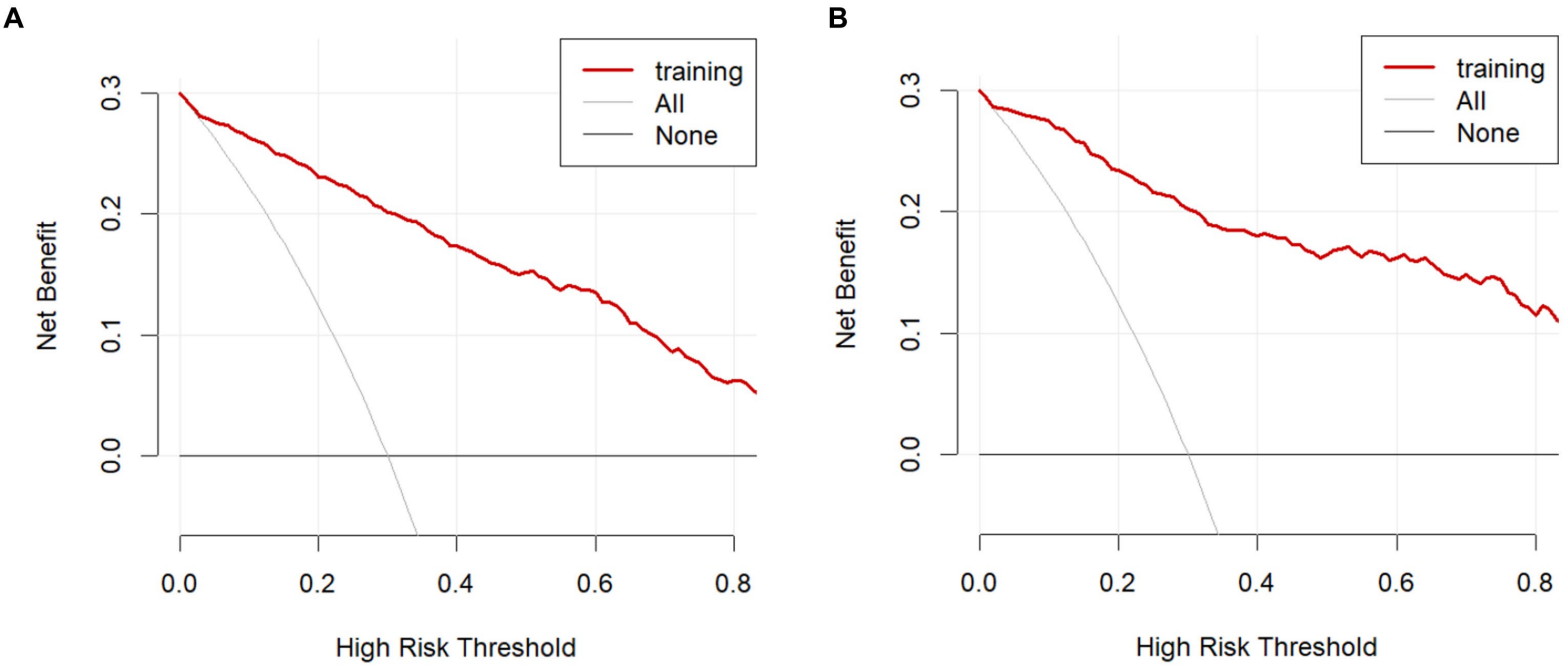
Decision curve analysis in the prediction of POF. **(A)** Training cohort. **(B)** Internal validation cohort.

The model was validated using a validation dataset. Its performance was assessed in terms of discrimination, calibration, and clinical utility. As illustrated in Figures 3A,B, the ROC curves for both the training and validation sets demonstrated the model’s capability to differentiate outcome events: the AUC for the training set was 0.924, and for the validation set, it was 0.941. The calibration curves in Figures 4A,B further supported the model’s validity, showing a close correlation between the predicted outcome events and actual occurrences. To further elucidate the clinical utility of the model, Decision Curve Analysis (DCA) was plotted in Figures 5A,B, indicating that our model provides a significant clinical net benefit. The results confirm that our model can accurately predict whether patients with AP will develop POF, underscoring its positive clinical implications. In addition, We validated our prediction model using the Hosmer-Lemeshow test and the coefficient of determination (*R*^2^). Hosmer-Lemeshow test on the internal validation cohort: The *p*-value was 0.086 (*p* > 0.05), indicating that there was no significant difference between the observed and predicted values. Coefficient of determination (R^2^) for the internal validation cohort: The *R*^2^ value was 0.546. These results indicate that our model demonstrates a good fit.

### Web-based calculator

**Figure 6:**
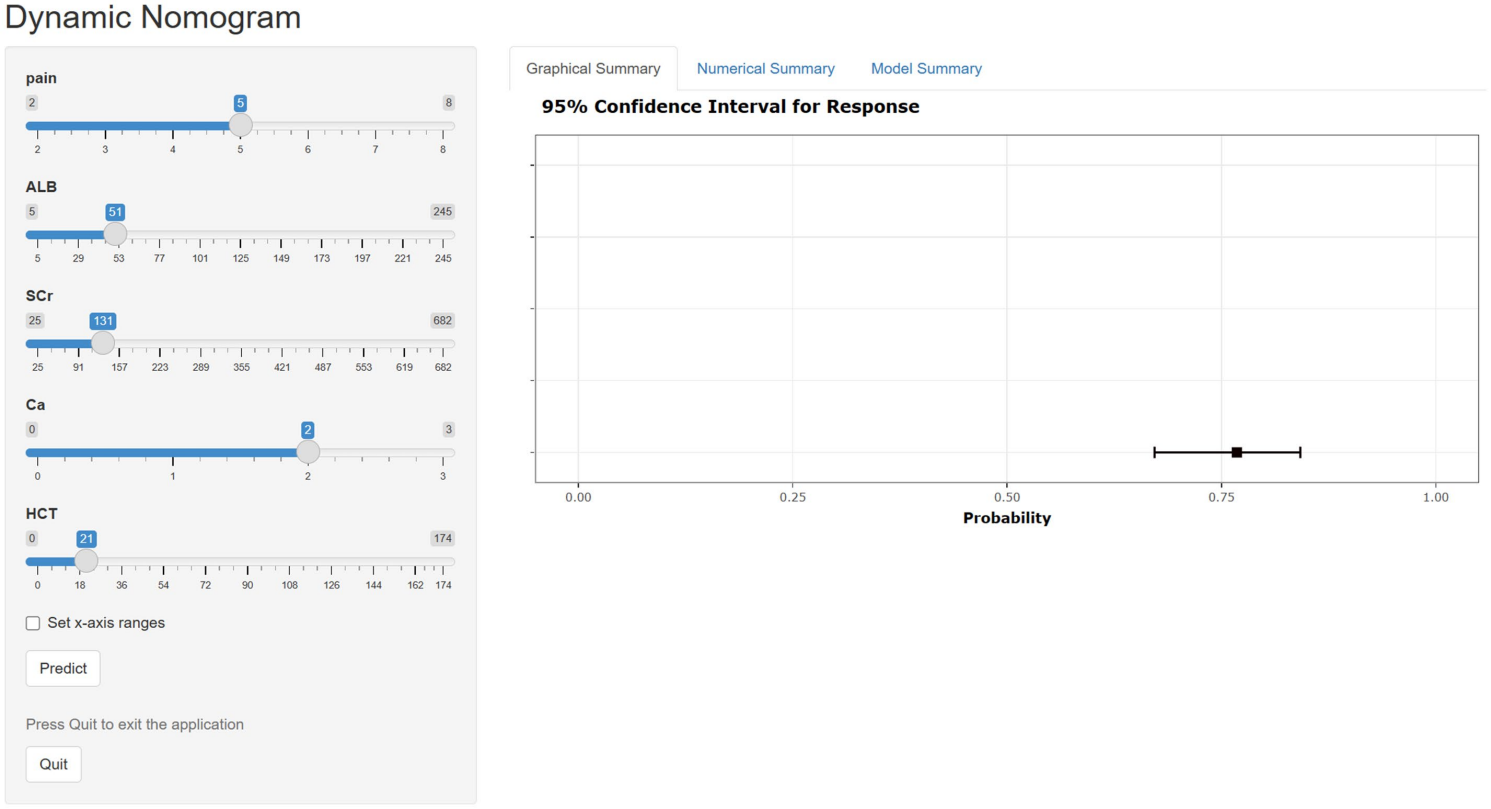
Dynamic web-based calculator for predicting POF in AP patients.

Although nomograms are intuitive, convenient, and cost-effective, they cannot provide precise numerical values during the calculation process. Therefore, we have developed a web-based calculator founded on nomogram principles to streamline the calculation process and obtain more accurate predictive values (https://xu-123.shinyapps.io/DynNomapp/). Select the appropriate variable values on the left, then click ‘Predict’. The probability of POF occurrence and the confidence interval will be displayed in the graph on the right. Please remember to click the ‘quit’ button after each use (Figure 6).

## Discussion

The occurrence of POF in conjunction with AP often signifies a poor prognosis for the patient. Studies indicate that organ failure lasting ≤48 h is usually associated with a lower risk of complications and mortality. However, patients with AP who develop POF lasting >48 h have a mortality rate as high as 50% (1). Early identification of risk factors associated with POF is crucial for the prognostic management of AP. The indicators collected in this study were obtained from the initial examination within 24 h of patient admission, minimizing interference from subsequent clinical treatments. Furthermore, we offer two visualization models for clinicians to choose from: a nomogram and a web-based calculator. The nomogram is straightforward, while the web-based calculator is convenient and precise. Our model has been validated and exhibits excellent predictive capabilities for AP with POF, facilitating early intervention by clinicians and reducing the mortality rate associated with POF.

Abdominal pain is an indispensable evaluation criterion in the diagnostic standards for acute pancreatitis. The latest prognostic scoring system for pancreatitis, the PASS system, also incorporates abdominal pain into its evaluation criteria (18). Pain is a subjective experience that is influenced by a variety of factors, both physiological and psychological. Additionally, there are individual differences in the understanding and perception of pain (19). Accurate and objective assessment of pain is crucial in clinical practice for the diagnosis and subsequent treatment of patients. At our institution, Assess using the Numeric Rating Scale (NRS) before implementing any pain management measures upon patient admission. Patients rate their pain intensity on a scale from 0 (no pain) to 10 (most severe pain). This scale can be rapidly implemented following training of medical staff and is considered the gold standard for pain assessment due to its speed, convenience, and low cost. The patient’s pain response is one of the most direct indicators of disease progression. Clinicians can evaluate the progression of a patient’s condition based on the clinical manifestations caused by the pain. Studies have shown that tolerable pain is associated with a favorable prognosis for the patient (20). Few studies have linked pain scores with adverse patient outcomes. This study concludes that pain scores at the time of admission are independent risk factors for the development of POF in patients with AP. This underscores the importance in clinical practice of not solely relying on laboratory test indicators but also incorporating the patient’s subjective experiences into the assessment of their condition, which aids in better evaluating the patient’s status and taking timely necessary actions.

ALB is an important biochemical marker for assessing a patient’s nutritional status and the severity of their illness. Since albumin has a half-life of 21 days, it may not be the optimal acute phase reactant. However, hypoalbuminemia is still associated with poor prognosis in various diseases. The occurrence of POF is often accompanied by a severe inflammatory response, leading to increased capillary permeability and the leakage of albumin from the vasculature into the interstitial space, resulting in the consumption of serum albumin. Patients with AP complicated by POF often have limited intestinal absorption capabilities, leading to inadequate protein intake. Additionally, the high metabolic changes within the body increase the demand for energy and protein, further reducing ALB levels. Studies have indicated that lower plasma albumin levels are associated with an increased number of complications, higher rates of subsequent infections, and increased mortality (21). Early low ALB levels are associated with poor prognosis in patients with AP (22). Patients with SAP who have higher serum albumin levels often have a better prognosis and subsequent quality of life (23). Hypoalbuminemia reflects poor nutritional status, enhanced inflammatory response, or reduced hepatic synthesis function, all of which may lead to a decreased resistance to disease and an increased risk of POF.Therefore, ALB levels can serve as an important indicator for assessing the severity of a patient’s condition and prognosis.

SCr is a widely recognized and commonly used renal function marker in the assessment of organ failure. This study indicates that serum creatinine can independently predict the onset of POF, consistent with the results of previous research (24). In patients with POF, the release of inflammatory signals from pancreatic acinar cells leads to the accumulation of fluid in the third space, resulting in renal ischemia. Concurrently, the influx of leukocyte interleukins, platelet-activating factors, and inflammatory mediators into the bloodstream exacerbates renal ischemia, causing sustained renal damage, which in turn leads to renal injury and associated increases in SCr (25). Studies have shown that acute kidney injury is often accompanied by organ failure in patients with AP, and the mortality rate in patients with acute kidney injury and AP exceeds 25% (26). Pete et al. pointed out that renal failure for more than 48 h in patients with SAP is a strong predictor of poor prognosis (27).

Serum Ca has been shown to be associated with clinical outcomes in pancreatitis (28). (1) Calcium ions can abnormally activate pancreatic enzymes, initiating autodigestion of the pancreas (29). (2) In the context of POF, specific calcium channels known as SOCE (store-operated calcium entry) are abnormally activated, facilitating the influx of calcium ions into cells and resulting in a decrease in serum calcium concentration. Concurrently, an excessive intracellular concentration of Ca^2+^ alters the permeability of mitochondria (30). The consumption of ATP is required to maintain toxic concentrations of Ca^2+^. Excessive accumulation of calcium ions within cells can disrupt the normal function of mitochondria, which may be a cause of pancreatic acinar cell death. Furthermore, calcium overload itself can trigger autodigestion and necrosis of pancreatic acinar cells (31). At the same time, fat necrosis in AP patients with POF can bind free calcium and further lead to hypocalcemia.

Studies have demonstrated that the HCT levels measured upon admission have the same sensitivity as the Ranson scores obtained after 48 h (32). Meanwhile,HCT is an independent risk factor for predicting mortality in AP (33). When admission HCT ≥44%, the incidence of POF reached 53.6% (34). This is similar to the results obtained in this study. Higher HCT values may be associated with inflammation and increased capillary permeability due to bradykinin-like substances. This can lead to peripheral vasodilation and the shift of fluid from the vasculature to the interstitial space, reducing circulating blood volume and relatively increasing the proportion of red blood cells.

In this study, we combined five selected predictive factors, each belonging to different systems: mental status, liver function, renal function, routine blood indicators, and pancreatic function. Consequently, this predictive model can comprehensively and accurately forecast the incidence of POF in patients with AP. Additionally, we have created visual nomograms and web-based calculators. These tools can be conveniently applied in clinical settings and provide valuable guidance for clinicians. However, the data for this study comes from a single center and employs a retrospective method, which has some limitations. In the future, validation should be conducted in multicenter, large-sample prospective studies.

## Conclusion

Pain score, serum creatinine, hematocrit, serum calcium, and serum albumin were independent predictors of acute pancreatitis complicated by persistent organ failure. A prediction model was developed based on these 5 clinical risk indicators and a nomogram and network calculator were constructed. The model had good prediction performance.

## Data availability statement

The raw data supporting the conclusions of this article will be made available by the authors, without undue reservation.

## Ethics statement

The studies involving humans were approved by the Shanxi Bethune Hospital (Ethical Approval Number: YXLL-2023-237). The studies were conducted in accordance with the local legislation and institutional requirements. The human samples used in this study were acquired from primarily isolated as part of your previous study for which ethical approval was obtained. Written informed consent for participation was not required from the participants or the participants’ legal guardians/next of kin in accordance with the national legislation and institutional requirements.

## Author contributions

JXi: Data curation, Formal analysis, Writing – original draft. MX: Data curation, Methodology, Writing – original draft, Writing – review & editing. JXu: Data curation, Formal analysis, Investigation, Writing – review & editing. JL: Investigation, Methodology, Writing – review & editing. FH: Data curation, Supervision, Writing – review & editing.
